# Antithrombin: Deficiency, Diversity, and the Future of Diagnostics

**DOI:** 10.1002/mas.21929

**Published:** 2025-03-15

**Authors:** Mirjam Kruijt, Christa M. Cobbaert, L. Renee Ruhaak

**Affiliations:** ^1^ Department of Clinical Chemistry and Laboratory Medicine Leiden University Medical Center Leiden The Netherlands

**Keywords:** antithrombin, diagnostics, mass spectrometry, medical test development, proteoforms

## Abstract

Our healthcare system provides reactive sick‐care, treating patients after symptoms have appeared by prescription of generic and often suboptimal therapy. This strategy brings along high costs and high pressure which is not sustainable. Alternatively, P5 healthcare is proposed focusing on five key elements: prevention, personalization, prediction, participation, psychocognition, however, changes in current clinical care pathways are required, for which antithrombin deficiency is a prime example. Hereditary antithrombin deficiency (ATD) is a genetic disorder, for which screening is instigated after a thrombotic episode. Current diagnostic tests for ATD lack sensitivity and refinement to correctly classify patients, and generic treatments are prescribed. A molecular understanding of ATD through a molecular diagnostic test that analyzes all clinically relevant features of antithrombin is required. Here, clinically relevant molecular characteristics of antithrombin, the diversity of antithrombin (deficiency) in heath and disease, and the strengths and weaknesses of antithrombin tests are reviewed. A mass spectrometry test that molecularly characterizes a patients antithrombin proteoforms harbors the highest potential to improve the clinical pathway for ATD. Application of this MS‐based test in a future enhanced clinical pathway will improve patient management and outcome through molecular characterization of antithrombin and enables the promise of P5 healthcare for ATD.

## 1 Introduction

Even though pleads have been made for the introduction of precision medicine, the current healthcare system is still based on reactive medicine in which patients first have to become ill and are subsequently treated with therapies tailored towards the average white male (Waldman and Terzic 2019). This healthcare system is not durable nor optimal and instead P5 (preventive, personalized, predictive, participatory, psychocognitive) medicine has been proposed as an alternative to reduce the disease burden in the general population and hamper the surge in healthcare costs (Honig and Terzic 2017; Waldman and Terzic 2018). Impact of P5 medicine is expected through prevention of disease, lowered time to accurate diagnosis, and optimized therapies in which response to therapy is increased (Pravettoni and Triberti 2020).

Laboratory medicine plays an essential role in the identification of diseases and patient management. Recently, the definition of laboratory medicine was established as: “a clinical science and discipline, devoted to the quantitative measurement, or qualitative assessment […] for either medical or research purposes. The results of these measurements are translated into actionable information for improving the care and/or maintaining the wellness of both a single individual and an entire population” (Lippi and Plebani 2020). The importance of laboratory medicine is clearly illustrated by a study finding that in a cardiology and oncology setting 66% of clinical decisions are affected by in vitro diagnostic (IVD) tests, with application of IVD tests in 88% of the initial diagnoses (Rohr et al. 2016). For medical tests to provide actionable information their test purpose should be clearly defined. This may entail setting a diagnosis, screening, risk stratification, and treatment selection and monitoring, highlighting the broad applicability of laboratory diagnostic tests (Hallworth et al. 2015). To facilitate the transition to P5 medicine, laboratory testing must be advanced to generate information of sufficient depth thereby enabling risk stratification and accurate patient diagnoses.

Current laboratory tests in diagnostic pathways for thrombotic diseases lack analytical and clinical specificity to provide information of sufficient depth to enable P5 medicine. Specifically, this also holds true for antithrombin deficiency (ATD) testing, and thrombophilia screening in general, where the utility of diagnostic tests is being questioned (Colucci and Tsakiris 2017; Hornsby et al. 2014). Several reasons have led to this skepticism: the relatively low prevalence of thrombophilia's, the lack of evidence that thrombophilia testing can prevent thrombosis in individuals lacking a history of thrombosis, and the limited effect of thrombophilia testing on treatment strategy (Cohn et al. 2012; Ashraf et al. 2019; Middeldorp 2016; Stevens et al. 2016). For ATD, this is further complicated by the questionable clinical performance of the current first line screening test to identify ATD (Orlando et al. 2015). As a consequence, this test is more often applied as a liver function biomarker, as antithrombin (AT) is produced by the liver, than for thrombophilia screening (Pereyra et al. 2017; Zhou et al. 2023). Because the first line AT test is not fit‐for‐purpose, personal and familial history and presence of factors provoking thrombosis play a large role in ATD diagnoses. It is known for ATD that thrombosis risk is heterogeneous and depends on the specific subtype (Luxembourg et al. 2014), which cannot be provided through anamneses. A laboratory diagnostic test that can differentiate low‐ and high‐risk patients would be required to optimize patient management by providing more accurate ATD diagnoses, guide (future) targeted therapies, and prevent recurring thrombotic events.

For a laboratory test to be successfully incorporated in a clinical care pathway, it must lead to proportionate clinical benefit. The European Federation of Clinical Chemistry and Laboratory Medicine (EFLM) has proposed a test evaluation framework (Horvath et al. 2014) in which five key components of in vitro tests are outlined. Ordered from development to clinical implications, the interrelated components are analytical performance, clinical performance, clinical effectiveness, cost effectiveness and broader impact. Newly developed tests should be evaluated for each of the five key components. Moreover, it is imperative to define the test role and test purpose of a newly developed test. To define the test role and test purpose as well as requirements for analytical and clinical performance of an AT test that enables P5 medicine it is imperative to understand the analytes molecular, biological, and clinical variation.

The aim of this manuscript is to compile the information required for the development of a next‐generation test for AT. It provides a narrative review of the aspects of AT and ATD relevant to understand and improve the clinical care pathway, current testing strategies for AT and ATD, and the application of mass spectrometry for molecular characterization of proteins. Lastly, we discuss the outlook of a novel mass spectrometry‐based test for molecular ATD diagnostics in the 21st century.

## 2 Deficiency

Antithrombin plays a pivotal role in the regulation of the coagulation cascade. The deprecated name for AT, antithrombin III, stems from the misunderstanding that multiple forms of AT exist, each with its own role in the coagulation cascade (Mosesson 2007; Egeberg 1965). Although further research revealed a single true AT protein, the idea of having multiple (proteo)forms of AT is still actual to date, albeit not based on varying roles, but due to the presence of mutant or atypical AT proteoforms. A minimal amount of functional AT is required to sustain life, and a complete deficiency is therefor lethal *in utero* (Ishiguro et al. 2000). However, AT proteoforms may have varying functionality. For instance, in healthy individuals already two proteoforms of AT exist, namely α‐AT and β‐AT, of which the main α‐proteoform accounts for ~90% of the total AT (Turk et al. 1997). However, mutations and posttranslational modifications give rise to many more proteoforms, as will be discussed later.

AT deficiency (ATD) is a clotting disorder caused by a low concentration and/or dysfunctional AT. Although often believed to have a fairly low prevalence rate between 1:2000 and 1:5000, these rates were based on assumptions rather than actual studies (Odegård and Abildgaard 1978; Bleich et al. 1975). The observed prevalence is in fact much higher in studies conducted in apparently healthy donors (1:400 to 1:600 (Tait et al. 1994); Wells et al. 1994; Tait et al. 1990; Ødegård et al. 1976), and is further increased in the venous thromboembolism (VTE) population (0.5 to 8:100 (Heijboer et al. 1990; Pabinger et al. 1992; Mateo et al. 1997; Miyata et al. 2009)). Of note, these prevalence rates were based on functional AT activity tests which are known to underdiagnose specific types of ATD and therefore true prevalence rates may exceed these numbers. ATD patients have the highest risks of thrombosis (odds ratio of 14 to 16) among inherited thrombophilia patients, highlighting the importance of correctly diagnosing and treating ATD (Di Minno et al. 2015; Croles et al. 2018). Patients are currently diagnosed as ATD by routine chromogenic AT activity tests, and even patients showing only a slight decrease of AT activity show an increased risk of thrombosis (Di Minno et al. 2014; Sokol et al. 2018). Furthermore, specific mutations in AT are known to have a pathogenic phenotype but do not lead to lowered activity test results (Orlando et al. 2015).

### 2.1 Molecular Characteristics and Structure of AT

**Figure 1:**
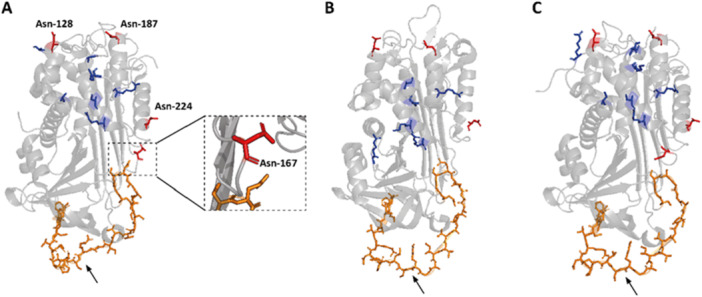
Molecular structures of antithrombin. (A) α‐AT monomeric cartoon representation with additional zoom‐in for Asn‐167 residue. (B) β‐AT monomeric cartoon representation. (C) Latent AT monomeric cartoon representation. Glycan positions are highlighted in red (displayed as sticks), heparin binding site in blue (displayed as sticks) and the reactive site in orange (displayed as sticks). The black arrow at the bottom indicates the cleavage target for proteases (the bond between Arg‐425 and Ser‐426). The X‐ray structures 1T1F (Johnson et al. 2006), 1E04 (McCoy et al. 2003) and 2BEH (Johnson et al. 2006) were retrieved from the PDB (Berman 2000) and the images were made with the PyMOL Molecular Graphics System (PyMOL). [Color figure can be viewed at wileyonlinelibrary.com]

The gene for AT, *SERPINC1*, encodes for 464 amino‐acids. This includes a 32 amino‐acid signal peptide which is essential for correct posttranslational processing and is cleaved off before secretion, resulting in a mature protein of 432 amino‐acids (Lane and Caso 1989; Fitches et al. 1998). Regarding posttranslational modifications, AT contains four N‐glycosylation sites (Asn‐128, Asn‐167, Asn‐187 and Asn‐224), of which one site (Asn‐167) naturally shows varying occupancy. The type of glycan attached at these sites is highly conserved, with 95% to 99% of the glycans being biantennary complex‐type glycans (Demelbauer et al. 2005). The main (glyco)proteoform of AT, α‐AT, is fully glycosylated, whereas the second form, β‐AT, lacks the glycan at position Asn‐167 (Turk et al. 1997) (Figure 1A,B respectively). The β‐AT proportion in plasma of healthy donors was found to lie between 5.9% and 10.7% (Römisch et al. 2002). Functionally, β‐AT has an increased affinity for heparin leading to higher functionality (Turk et al. 1997). This may be explained by the glycan interfering with the heparin‐induced conformational change of α‐AT from the inactive to the active form, or alternatively by steric hindrance of the heparin binding site by the large glycan moiety (Turk et al. 1997). Levels of β‐AT are elevated in young children, which is suggested to compensate for lower overall AT concentrations in infants (Karlaftis et al. 2014; Ignjatovic et al. 2010a). Furthermore, β‐AT was found to play a key role in protection from vascular injury in a rabbit model (Witmer and Hatton 1991; Frebelius et al. 1996). Recently, a study showed that β‐AT may also play a role in ameliorating certain types of ATD as mutant β‐proteoforms, in contrast to mutant α‐proteoforms, retain their functionality despite the presence of HBS mutations (Martínez‐Martínez et al. 2012). Thus, β‐AT appears to have high clinical relevance, even though this proteoform is currently not specifically evaluated by diagnostic tests.

A second naturally occurring proteoform has been suggested to be of clinical relevance: latent AT, a conformer of native AT (Figure 1C). Latent AT is characterized by insertion of the reactive center loop into the A‐β‐sheet, leading to an irreversible inactive state which is unable to form a complex with a target protease, FIIa, and has low heparin affinity (Wardell et al. 1993). The latent proteoform is highly thermostable (with melting temperatures of up to 127°C compared to 60°C for native AT) and resistant to denaturation by high concentrations of urea (Wardell et al. 1997; Mushunje et al. 2004). This proteoform was found to circulate in healthy donors at highly stable percentages of around 2.6% ± 0.9% of the total AT pool (Mushunje et al. 2004), likely owing to the metastable conformation of native AT resulting in natural formation of the latent proteoform. Increased levels of the latent proteoform have been found during sepsis (Mushunje et al. 2004), and in patients with specific mutations in AT, such as p. Met283Val, p. His401Tyr, p. Pro439Thr, and p. Pro461Ser (de la Morena‐Barrio et al. 2017). As slight increases in the temperature may facilitate the transition to the latent proteoform, the presence of increased levels of latent AT during fever may lead to thrombosis, both in ATD patients with mutations leading to high latent AT levels as well as in the general population (Mushunje et al. 2004; Corral et al. 2005).

Serpins, the superfamily of proteins to which AT belongs, all have a similar structure containing a reactive site which determines their substrate specificity (Lane and Caso 1989). For AT, this site is positioned from Gly‐411 to Asn‐430 with the bond between amino acids Arg‐425 and Ser‐426 being the cleavage target of proteases (Rezaie and Giri 2020). Cleavage of this site is followed by a conformational change in AT leading to the inactivation of the protease (Björk et al. 1982). Besides the reactive site, AT contains a heparin‐binding site which involves positively charged residues, lysines and arginines, located across the AT molecule (Ersdal‐Badju et al. 1997). Interestingly, the length of the heparin polysaccharide influences the affinity of AT for specific proteases (Bray et al. 1989). Furthermore, the mechanism by which the two main target proteases, Factor Xa and FIIa, interact with AT also differs. Whereas the affinity of FXa is highly dependent on the heparin dependent conformational change in AT, FIIa relies mostly on a so‐called “template” effect in which heparin acts as a scaffold for the AT‐FIIa‐heparin complex to form (Streusand et al. 1995; Olson et al. 1992).

### 2.2 Subtypes of Antithrombin Deficiency

Hereditary ATD is subdivided into two main types, type I ATD, encompassing quantitative deficiencies, and type II ATD, encompassing qualitative (functional) deficiencies, both caused by mutations in the *SERPINC1* gene (Patnaik and Moll 2008). Currently, over 350 different mutations in AT are known, the majority being point‐mutations (Corral et al. 2018; Human Gene Mutation Database 2021). For patients with type I ATD, the mutation is always present in a heterozygous state and hinders the translation and/or secretion of the mutant proteoform leading to reduced concentrations of wildtype AT being present in circulation compared to healthy individuals. Type II ATD is caused by mutations that affect the functionality of the protein but do not (fully) omit translation and secretion, leading to a mixture of wildtype and variant proteoforms present in circulation for heterozygous mutations or exclusively variant proteoforms present for homozygous mutations. Similar to type I ATD, type II ATD mutations are mostly present in a heterozygous state, likely caused by the detrimental clinical effects of homozygosity (Ishiguro et al. 2000). However, a small number of type II mutations with mild phenotypes in the heterozygous state may be present in homozygous state (such as p. Leu131Phe also known as AT Budapest III, or p. Arg79Cys also known as AT Toyama) and often lead to severe clinical phenotypes.

Beyond mutations in the *SERPINC1* gene, ATD may also be caused by aberrant or missing glycosylation, for instance caused by congenital disorders of glycosylation (CDG) (de la Morena‐Barrio et al. 2016). CDGs are a collection of genetic disorders affecting the overall glycosylation machinery and impacting many glycoproteins including AT (Jaeken and Matthijs 2001). Furthermore, specific mutations in AT have been associated with hypoglycosylation, namely p. Asn224His and p. Glu227Lys, which affect the N‐glycosylation site at position Asn‐224. Patients with these mutations presented with early and recurrent thrombosis, even though AT activity tests showed normal results (de la Morena‐Barrio et al. 2022a). With many variations possible in AT, it is not surprising that the clinical phenotype of ATD is highly heterogeneous, and that identifying AT deficiency can be challenging for certain subtypes.

## 3 Current Clinical Pathway

### 3.1 Recommended Testing Strategy

Due to the complexity of ATD, there is no single test available that analyzes all clinically relevant aspects of AT, namely its activity, concentration, and molecular characteristics. Instead, various diagnostic tests are available, each analyzing an individual aspect of AT. A guideline on the diagnostic pathway was established by the Scientific and Standardization Committee of the International Society on Thrombosis and Haemostasis (ISTH) which recommends a combination of activity and antigen tests in the diagnosis and subtyping of hereditary ATD (Van Cott et al. 2020). The guideline, in agreement with general practice, recommends to first perform an activity test, as this test identifies (most) type I as well as type II ATDs. Activity tests may be influenced by interfering compounds (such as anticoagulants) and under certain conditions, thus repeat testing is required to exclude a transient state of ATD. To determine the subtype of ATD, a low activity result should be followed up by an antigen test. Low results on both tests indicate a type I ATD, while low activity and normal or slightly decreased antigen levels indicate a type II ATD. Of note, the ISTH guideline does not state when the testing strategy may be applied, which is a controversial topic and will be discussed later (‘Variation in guidelines’). Taken together, the current strategy for identifying ATD is a rather blunt approach that oversimplifies the complexity of ATD, does not provide molecular insight, nor facilitates a basis for precision diagnostics and treatment.

### 3.2 Activity Tests

Being the first‐line test for diagnosing ATD, a wide range of commercial CE‐IVD marked AT activity tests are available from different manufacturers. The tests assess the functionality of the total AT pool present in patient plasma. All tests have a similar design, with only minor changes since the development of AT activity tests in the 1970s (Blombäck et al. 1974); citrate plasma is mixed with an excess of AT target protease, forming a complex with AT, after which the remaining target protease cleaves a chromogenic substrate leading to a color‐reaction (Muszbek et al. 2010). Depending on the substrate a specific optical density is measured, the height of which is inversely correlated with the amount of AT present in the sample. To interpret the OD, it is suggested to generate a calibrant based on a large pool of healthy donors (*N* ~ 100) and set the value thereof at 100% activity. Although small fluctuations occur between laboratories, values between 80% and 120% are generally considered healthy. The test is easy to use and can be applied on automated coagulation analyzers, enabling a rapid and inexpensive means of screening for ATD.

The various activity tests employ varying target proteases, either FIIa or FXa, of varying origin, either recombinant human or bovine. The use of either FIIa or FXa as a substrate may influence the sensitivity for specific types of ATD (Kovács et al. 2013), as the mechanism by which AT inhibits these two target proteases also differs, as described previously. The target proteases added in the test can also be inhibited by other anticoagulant proteins to which end heparin is added to increase the functionality of AT and make the test more specific (Cooper et al. 2011; Moore 2024). As mentioned earlier, heparin acts as a co‐factor for AT, greatly increasing its activity, and heparin chain length determines specificity of AT for the target protease. Heparin content in the various kits differs per activity test and the type of heparin is often not specified.

**Figure 2:**
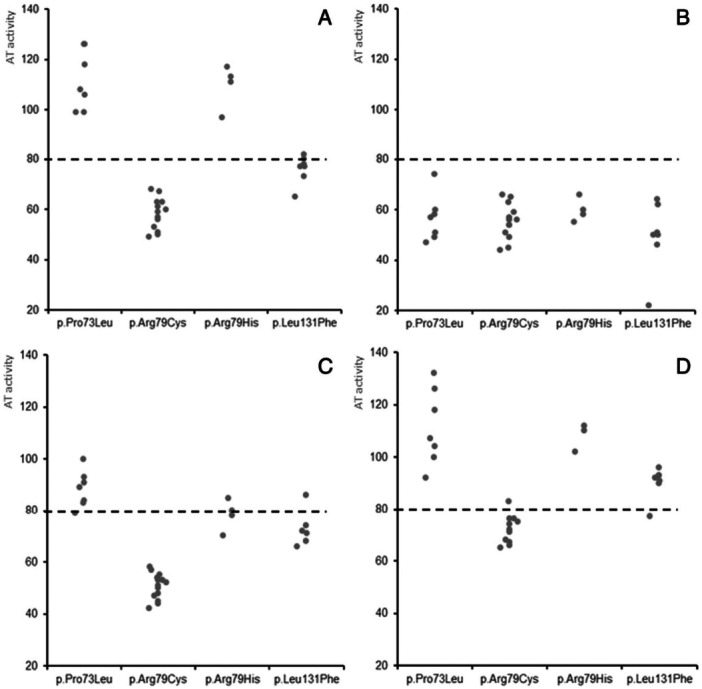
Antithrombin activity in 4 different heparin binding site mutations. (A) HemosIL® Liquid AT, (B) Innovance® AT, (C) Coamatic® AT, and (D) Biophen® anti‐IIa. Dashed line represents lower limit of normal range. Reprinted with permission from Orlando et al. (2015) (Orlando et al. 2015).

The variability between test kits leads to varying results depending on which kit a laboratory applies. Furthermore, the different subtypes that occur in ATD also behave differently within and between kits, depending on the specific mutation (Orlando et al. 2015; Kovács et al. 2013). It is therefore no surprise that the diagnostic sensitivity of the activity test differs between manufacturers and specific mutations. A study by Orlando et al. clearly demonstrated this by comparing the results of four different AT HBS mutations using four different activity tests (Figure 2) (Orlando et al. 2015). In this study only a single activity test (test B) could identify all four ATD HBS mutations tested. Similar results have been found in other studies (Kovács et al. 2013; Ungerstedt et al. 2002; Kristensen et al. 2007; Liao et al. 2022; Yu et al. 2022), prompting the question whether the current testing strategy relying solely on activity tests as a means of diagnosing ATD is appropriate, or if alternative (molecular) options are required. Of note, good long‐term performance of the activity tests was found in external quality assessment programs, with medians of 7.2% and 9.4% for the within‐ and between‐laboratory CV, respectively (Meijer et al. 2003). Such programs require large volumes of sample to supply all participating laboratories, entailing that the sample is often comprised of a large pool of donors and the level of the analyte of interest is artificially altered. Thus, these samples at best reflect the performance of activity tests for type I ATD, whilst the more problematic performance for molecularly heterogeneous type II ATD samples remains unexplored.

### 3.3 Antigen Tests

Once ATD is diagnosed using the activity test, the antigen test may be employed as a follow‐up test for low activity samples to classify the ATD subtype. Antigen tests provide a (relative) concentration thereby allowing for discrimination between quantitative type I and qualitative type II ATD deficiencies. There are various CE‐IVD marked diagnostic tests to determine AT antigen, based on immunonephelometric or (latex) immunoturbidimetric principles. This entails that antibody against AT is mixed with patient sample and the formed immune complexes containing AT are measured by light scattering or light intensity. Therefore, these tests rely “blindly” on the antibody employed, offer no molecular information, and consequently do not discriminate between AT proteoforms (or interferents). The readout of the antigen tests is often stated in units or % and are therefore by no means an absolute indication of the concentration of AT. To indicate the ATD subtype, one may divide the relative antigen result over the relative activity result and observe if an abnormal ratio is found compared to established reference intervals for both subtypes (Cooper et al. 2011; Mitsuguro et al. 2010). Alternatively, one may interpret both values separately using their respective reference intervals thereby discriminating type I ATD, having below average values for both tests, from type II ATD, having normal values only for the antigen test. The first method is of course more sensitive in the correct identification of type II ATD mutations that may have both a qualitative and a (minor) quantitative defect. Of note, this discrimination between type I and type II ATD may be too simplistic considering new insight generated into ATD showed that there are also type II ATD with a quantitative effect, as well as type I ATD with a qualitate effect, as discussed later in “Multiple‐reaction monitoring mass spectrometry.” However, as AT subtyping does not necessarily influence patient management, antigen testing is only applied scarcely.

## 4 Sources and Effects of Diversity in Antithrombin

Thus far, the diversity of the molecular features of AT has been discussed, whilst conversely the current diagnostic pathway for ATD is based on a simplified view that we may diagnose ATD in patients harboring varying molecular AT proteoforms by merely examining the overall activity of the total AT pool. To improve the diagnostics of ATD we must be aware of all aspects influencing AT levels. Therefore, the next section provides an overview of additional sources of AT diversity that can affect ATD tests and the performance of these test in the ATD clinical pathway.

### 4.1 Biological Variation

Naturally, all components of the blood (or any biological fluid) have a fluctuating level, for instance due to changes in the production or consumption, cyclical influences, or because they are influenced by other biological processes (Badrick 2021). For a clinical laboratory test to discriminate between a healthy individual and a patient harboring a disease, it is essential to know the variation of the analyte of interest in a healthy population, the so‐called biological variation (Sandberg et al. 2023). Specifically for the development of a diagnostic test, this information is necessary to establish the required analytical performance.

Many factors have been reported to affect the concentration and/or functionality of AT. Age is a known factor influencing the levels of AT (Monagle et al. 2006; Weidhofer et al. 2018; Toulon et al. 2016). During the first year of life hepatic immaturity leads to reduced activity levels of AT, with studies reporting values in neonates as low as 33% (Toulon et al. 2016). These levels reach normal values after 1 year, although studies have found that throughout childhood and adolescence, higher interindividual variation in AT activity values is found, although one study found the absolute values to be lower in children than in adults while two studies showed inverse results. Beyond activity levels, Ignjatovic et al. demonstrated that the molecular composition of AT is also different at a young age, with an increased contribution of the β‐proteoform to the overall AT activity (Karlaftis et al. 2014; Ignjatovic et al. 2010b).

Concerning gender, multiple studies have investigated the difference in AT activity between men and women with conflicting results. However, the most recent studies (published in the last 15 years) did observe significant differences (Franchi et al. 2013; Tabibian et al. 2022; von Falckenstein et al. 2022). These studies reported a lower level of AT activity in women of reproductive age compared to men, although the absolute difference was often not more than 2% to 3%. Interestingly, whereas the activity values of women tend to increase throughout life, the levels in men start to decrease around 40 years of age leading to men having lower values of AT activity than women at higher ages, with observed absolute differences of 4% to 9%. This gender difference may be attributed to the influence of hormones, all the more as it is known for women that hormones influence AT activity. For example, oral contraceptive use is a known hormonal factor influencing AT activity (Van Cott et al. 2020). Throughout life, women taking oral contraceptives have lower AT activity values of around 3% to 4% (Franchi et al. 2013; Tabibian et al. 2022). Of note, this does not explain the lower AT activity values between men and women of reproductive age, as a difference was also found when comparing men to women not on contraceptives (Tait et al. 1993). After menopause, AT activity levels are around 2% to 3% higher than in pre‐menopause women. In pregnant women, it was found that AT activity levels lower approximately 10% to 20% during the course of pregnancy (Kristoffersen et al. 2017; James et al. 2014).

Taking these variations into account, it was suggested to establish specific reference intervals for age and gender groups (Weidhofer et al. 2018; Tabibian et al. 2022). However, beyond the notion that AT is decreased in neonates and pregnant women, adjusted reference intervals are not common practice in clinical laboratories. Interestingly, a study by Goldman‐Mazur et al. (2019) found a significant increase in the detection of ATD in men suspected of having a thrombophilia (due to clinical indication) aged > 50 years versus men < 50 years (Goldman‐Mazur et al. 2019). This could either indicate that the decreased levels of AT found in older men lead to an increase of clinical events, or that ATD is overdiagnosed in this specific group. Although the observed absolute differences in age and gender were not major in adults (the largest observed difference was 102.2% activity for males vs. 111% activity for females aged 55–59 (Tait et al. 1993)), specifically for borderline AT activity values these differences could lead to over‐ or underdiagnosis.

Although this more concerns the prevalence of ATD, it is important to note that ATD prevalence may differ depending on ethnicity and nationality. For example, a study reported an increased prevalence of ATD in VTE patients among Thai, Chinese and Japanese ethnic groups compared to Caucasians (Angchaisuksiri et al. 2007). Furthermore, it is known that certain mutations occur in high prevalence in specific regions, such as AT Basel in Finland and AT Budapest III in Hungary (Puurunen et al. 2013; Gindele et al. 2016).

Biological variation may lead to differences in the analytical performance required for a diagnostic test. According to the Milan consensus, diagnostic tests should comply with predefined analytical performance specifications which, ideally, should be based on outcome studies assessing the effect of analytical performance on patient outcome (Sandberg et al. 2015). However, as these are not available for ATD, the alternative is to use biological variation data to establish these specifications. Fortunately, the European Federation of Clinical Chemistry and Laboratory Medicine (EFLM) working group on Biological Variation maintains a database containing information on the biological variation observed in laboratory measurands (Aarsand et al. 2024). For both the AT activity (Table 1) and AT antigen test (Table 2), biological variation data is available, including the observed variation within‐ and between‐subject (CVi and CVg, respectively), allowing calculation of the total allowable error of these the tests.

**Table 1:** Biological variation values of the AT antigen test as published by the EFLM Biological Variation Database.

Specification	CVi	CVg	Imprecision (CVa)	Bias	MAu	Total error
Minimum	7.2	5.0	5.4	3.3	10.8	12.2
Desirable			3.6	2.2	7.2	8.1
Optimal			1.8	1.1	3.6	4.1

*Note:* Values were based on based on (Thompson et al. 1987). Accessed 28th of June 2024 (Aarsand et al. 2024).

Abbreviations: CVa, analytical variation; CVg, between‐subject variation; CVi, within‐subject variation; Mau, maximum allowable measurement uncertainty.

**Table 2:** Biological variation values of the AT activity test as published by the EFLM Biological Variation Database.

Specification	CVi	CVg	Imprecision (CVa)	Bias	MAu	Total error
Minimum	3.4	7.8	2.5	3.2	5.1	7.4
Desirable			1.7	2.1	3.4	4.9
Optimal			0.8	1.1	1.7	2.5

*Note:* Values were based on based on (Kristoffersen et al. 2017; Thompson et al. 1987; Costongs et al. 1985; Chambless et al. 1992; Blombäck et al. 1992; de Maat et al. 2016; Aarsand et al. 2021; Wada et al. 2004; d'Eril et al. 1997). Accessed 28th of June 2024 (Aarsand et al. 2024).

Abbreviations: CVa, analytical variation; CVg, between‐subject variation; CVi, within‐subject variation; Mau, maximum allowable measurement uncertainty.

### 4.2 Pathologic Variation

Beyond biological variation found in the normal population, several pathological states also bring along variation in AT. For instance, there is a clear link between low activity values and liver diseases such as liver cirrhosis, liver carcinoma, and hepatitis, which can be explained by the liver being the site of production for many coagulant factors including AT (Chan et al. 1979; 1981; Kłoczko et al. 1992; Papatheodoridis 2003; Knot et al. 1984). Furthermore, fever may lead to a reduction in AT activity as lowered levels were found during bacterial and viral infections (Forsblom et al. 2019; Kale et al. 2010; Abdollahi et al. 2011; Wiersinga et al. 2008) as well as during sepsis (Ding et al. 2013), potentially explained by an increase of the latent AT proteoform (HernÁNdez‐Espinosa et al. 2007). Importantly, liver diseases, infections and sepsis may also lead to lower AT levels due to overconsumption of AT as a results of disseminated intravascular coagulation (DIC) (Adelborg et al. 2021). Furthermore, various pathologic aspects have shown contradicting results. Obesity has often been linked to lowered AT activity values and increased incidence of ATD (Bilge et al. 2012; Batist et al. 1983; El‐Menyar et al. 2018; Overby et al. 2009), although contradicting studies showed inverse or insignificant results (Kornblith et al. 2015; Holländer et al. 2015; Baena‐Fustegueras et al. 2013). Similar conflicting results were found for both type I and type II diabetes mellitus (Reverter et al. 1997; Ceriello et al. 1986; Barillari et al. 2009; Hughes et al. 1983). However, this does indicate that caution should be taken when interpreting activity results from patients with metabolic disorders. Bowel disorders such as ulcerative colitis, irritable bowel disease and Crohn's disease have also been mentioned to alter AT activity values although once again there are conflicting reports stating increased, decreased, or similar levels in patient groups versus controls (Kraiem et al. 2016; Alkim et al. 2011; Bernhard et al. 2011; Senturk et al. 2010).

Although not directly caused by a disease state, various medications may also alter AT levels. Reports have mentioned an effect of heparin therapy, asparaginase and antipsychotics (Levy et al. 2000; Liebman et al. 1982; Carrizo et al. 2008). Furthermore, direct oral anticoagulants (DOACs) are known to influence results of AT activity tests, which, depending on the DOAC and the factor in the test, may lead to increased results (Adcock and Gosselin 2015). It is therefore critical that in patients taking DOACs samples are taken after clearance of the medication, which may be challenging if patients are on anticoagulation due to a thrombotic incident, or that reversal agents are used before testing (Ząbczyk et al. 2021; Vercruyssen et al. 2023).

### 4.3 Clinical Symptoms: Thrombosis

Variation in biological and pathological states lead to differences in AT activity values, but conversely the molecular diversity in AT also leads to variation in the clinical effects of ATD. Venous thromboembolism (VTE) is a well‐established pathogenic consequence of low quantities and/or dysfunctional AT, with a 14 to 16 times increased risk compared to non‐deficient individuals (Di Minno et al. 2015; Croles et al. 2018). In fact, the high occurrence of VTE in a single family gave the first indication that thrombosis risk could be caused by a genetic factor (Egeberg 1965). The co‐occurrence of low AT activity in the same family linked ATD to thrombosis. However, although in general ATD leads to a high risk of VTE, this is heavily influenced by several factors such as environmental factors, additional genetic variation leading to pro‐ or antithrombotic states and by the ATD subtype. Type I ATD is known to lead to a high risk of VTE, with reports indicating that 60% to 86% of patients suffer from VTE (Luxembourg et al. 2014; Mitsuguro et al. 2010; Alhenc‐Gelas et al. 2017). Type II ATD is generally considered to be more benign, although one study found type II RS as well as type II PE ATD to show risks similar to type I ATD (Croles et al. 2018). A second study even found a higher risk of (spontaneous) VTE in type II RS ATD patients compared to type I ATD patients (Alhenc‐Gelas et al. 2017). The overall lower risk of type II ATD is thus mainly attributable to type II HBS, although within this subtype high heterogeneity between specific causative mutations is observed. Many mutations are linked to type II HBS ATD and combined with the relatively low prevalence of ATD (and perhaps the difficulty to identify type II HBS using current diagnostics) this hampers the inclusion of ATD patients with a specific mutation in studies for in‐depth characterization of the risks of individual HBS mutations. Consequently, only a few studies could recruit sufficient patients with a similar genetic background to analyze mutation specific risks of thrombosis. These studies found VTE occurrence in patients with type II HBS mutations to range between 0% and 50% for heterozygous mutations, even rising to 88.8% for the homozygous type II HBS Budapest III mutation (Luxembourg et al. 2014; Puurunen et al. 2013; Alhenc‐Gelas et al. 2017; Gindele et al. 2021).

### 4.4 Clinical Symptoms: Alternative Clinical Phenotypes

Although there is an undisputable link between ATD and VTE, additional clinical implications of ATD have been reported but remain controversial due to lack of evidence. For instance, arterial thromboembolism has been reported to occur in increased frequencies in patients with specific mutations, such as AT Basel (p. Pro73Leu) (Gindele et al. 2021), AT Cambridge II (p. Ala384Ser) (Roldán et al. 2009), and AT Padua (p. Arg79His) (Orlando et al. 2015). A meta‐analysis investigating the risks of ATD in general reported an increased OR of 1.25 for arterial ischemic stroke (Chiasakul et al. 2019), although this was not found to be significant likely caused by the lack of discrimination between subtypes or mutations. In contrast, a meta‐analysis investigating the risk of arterial ischemic stroke in a pediatric population with thrombophilia found a significant OR of 3.29 for ATD (Smith et al. 2021a; Kenet et al. 2010). Thus, although controversial, arterial complications should not be disregarded in the context of ATD.

Recurrent pregnancy loss and obstetric complications are often mentioned to be related to ATD. Both type I and type II ATD were found to lead to high risks of obstetric complications (Puurunen et al. 2013; Kovac et al. 2019; Kruijt et al. 2022; Abbattista et al. 2020; Roberts et al. 2022), with increased attention to specific mutations such as AT Basel (p. Pro73Leu) and AT Budapest III (p. Leu131Phe). As expected, as pregnancy is a provoking factor, studies describe a high incidence of thrombosis in pregnant women with ATD, with observed VTE occurrence ranging between 10% and 33% depending on the mutation and whether the mother has a history of VTE. This is in line with a previous finding that in 12.0% of the women experiencing VTE during pregnancy ATD was diagnosed (McColl et al. 1997), indicating that ATD plays a major role in pregnancy associated VTE. Moreover, an increased incidence of obstetric complications such as placental abruption or (late) pregnancy loss was also reported, with complication rates between 29.4% and 50%, once again depending on the mutation and exacerbated in mothers who had previously experienced VTE. However, similar to arterial complications, the majority of evidence is limited to small studies. In an attempt to come to a strong conclusion, systematic reviews have tried to pool the relatively larger studies (often still comprising of less than 100 participants with various thrombophilia's). These reviews do indicate an effect of thrombophilia on pregnancy outcome, although they still state that evidence is too weak to provide strong recommendations (Robertson et al. 2006; Alfirevic et al. 2002). Therefore, current guidelines advise against thrombophilia screening in women experiencing recurrent pregnancy loss (Youssef et al. 2019). Similarly, treatment strategies for women with thrombophilia experiencing obstetric complications are controversial, with small case studies reporting favorable outcomes while larger studies, lacking patient stratification, advise against the use of prophylaxis to improve pregnancy outcomes (Pabinger 2009; Quenby et al. 2023).

Taken together, the assumption that ATD is merely involved in the development of thrombosis is unlikely and if there is one thing that all studies agree on, it is that more research (and better research methods) into ATD and thrombophilia's in general is crucial.

### 4.5 Variation in Guidelines

Clinical pathways for hereditary thrombophilia's, as is the case for ATD, suffer from a low amount of evidence to guide clinical decision making. For ATD specifically, this is largely attributed to the fact that larger studies on (hereditary) thrombophilia's do not include sufficient patients with ATD to generate strong evidence concerning ATD. Furthermore, these studies identify patients based on general screening tests for thrombophilia's such as the AT activity test, which is known to miss certain subtypes of ATD and does not give molecular insight. This leads to an underrepresentation of ATD in general as well as a low variety of ATD subtypes in studies on which guidelines are built. Furthermore, as described previously, “the general ATD patient” does not exist when reflecting on the large variation found between ATD individuals. Thus, generalized approaches for the screening of all persons suspected of having ATD or a general treatment for ATD patients is likely not appropriate.

However, in our current healthcare system, even if evidence is lacking, guidelines are still being established. The most recent American Society for Hematology (ASH) guideline on thrombophilia testing is comprised of 23 recommendations, of which only 1 was a strong recommendation and all others are stated as suggestions due to low amount of available evidence (Middeldorp et al. 2023). The British Society for Hematology also provided a specific guideline on recommendations for when testing a deficiency of natural anticoagulants, such as AT, is warranted. This guideline stated to only test if clinical management would be impacted although, similar to the ASH guidelines, the level of certainty in the evidence is low (Arachchillage et al. 2022). Similar issues are found in the treatment of VTE, with major guidelines providing, sometimes conflicting, recommendations on the (prevention and) treatment of VTE in the context of surgery or pregnancy based on weak evidence for the majority of recommendations (Bates et al. 2018; Anderson et al. 2019).

Guidelines provide recommendations based on the general patient and cost effectiveness of testing and treatment. In contrast, individual clinicians are inclined to test patients or family members in situations not recommended by these guidelines or treat them based on their own clinical experience on ATD patients (Marongiu et al. 2024; Pabinger and Thaler 2019; Hart et al. 2022). Varying views on the value of a thrombophilia diagnosis has led to varying clinical pathways for screening in institutions diverging from major guidelines such as the ASH guideline (Marongiu et al. 2024; Djulbegovic et al. 2024; Jackson et al. 2008). Beyond the views of individual clinicians, the concept of P5 healthcare entails empowerment of our patients and giving them the right to know about their health status, thereby transforming them from bystanders to active decision‐makers (Prodan Žitnik et al. 2018). Thus, if we want to transform the ATD clinical pathway to a P5 healthcare approach, we must generate more information on the individual patient‐level, instead of focusing on generalized concepts of ATD or thrombophilia.

### 4.6 Influence of Diversity on the Current Clinical Care Pathway

**Figure 3:**
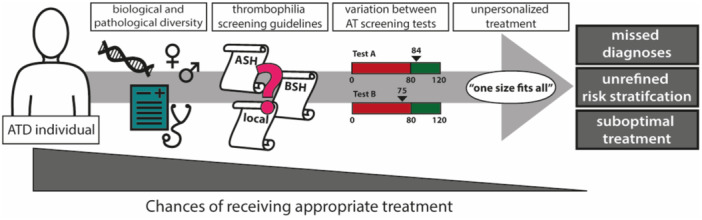
Overview of the influence of components of diversity on the clinical pathway for ATD patients. ATD patients are inherently diverse due to genetic, biologic, and pathological factors. Furthermore, the decision on when to screen for thrombophilia's differs depending on the guideline a healthcare setting adheres to, or the clinicians own interpretation. If screening is employed, diversity of screening tests may lead to missing diagnoses, depending on which test is employed. Lastly, even if patients are correctly diagnosed, the current treatment strategy is still a generalized anticoagulant treatment without considering the molecular basis of the ATD. Together, this diminishes the chances that ATD patients are treated appropriately, and VTE recurrence risk or overtreatment of the ATD individual may persist. [Color figure can be viewed at wileyonlinelibrary.com]

If one thing is clear from the currently observed and oftentimes unexplained diversity in AT and ATD, it is that fifty years of AT research employing mostly the functional AT activity test has not provided us the evidence to sensitively and specifically diagnose ATD patients, prevent VTE (or other disease) occurrence nor treat patients optimally. The scraps of evidence on which current guidelines and treatments are based on has led to an unrefined clinical care pathway (Figure 3). To improve this pathway, we must better understand AT and ATD, and find approaches to implement this new knowledge in clinical care.

## 5 Alternative Testing Options

**Figure 4:**
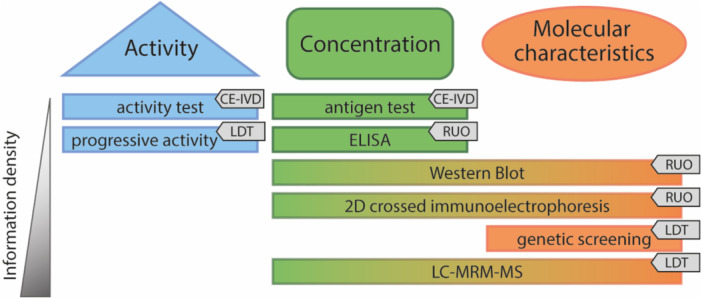
Test options for AT. Overview of available options to investigate AT, categorized by which aspect of AT the test focuses on. CE‐IVD, conformitee européenne in vitro diagnostics; LDT, laboratory‐developed test; RUO, research use only. [Color figure can be viewed at wileyonlinelibrary.com]

Most studies into ATD have employed AT activity tests, as these are commercially available and easily applied in large cohorts. However, the low level of information gained from activity tests hampers stratification of patients in these cohorts, nor do they identify all patients with ATD. To this end, specialized research into AT has often resorted to alternative techniques that are more labor intensive but provide additional information on for instance the heparin affinity or glycosylation status of certain proteoforms (Figure 4). Although these techniques offer valuable information in the research setting, their translation to patient diagnostics is often not possible, limiting their usefulness. Only two techniques have succeeded in progressing from research use only, to their applicability in patient diagnostics, namely genetic screening, and LC‐MRM‐MS analysis. An overview on all techniques, alternative to the standard activity and antigen test, currently applied in AT research and (if possible) diagnostics will be provided in this section to provide an overview of the strengths and weaknesses of each test and generate insight into which (molecular) aspects of AT can and should be investigated.

### 5.1 Research‐Only Methods

In the research setting, multiple techniques are available to investigate AT, as there are often no stringent constraints on analytical performance or sample throughput. Historically, 2D crossed immunoelectrophoresis (using heparin in the first dimension) was often applied to investigate the AT concentration of patient samples, enabling relative quantitation of AT (Sas et al. 1975a). The addition of heparin in the second dimension of the gel enabled exploration of the heparin affinity of the AT proteoforms in a sample and increased the information density of the test. Although labor intensive, the relatively simple execution and ability to gain functional information entails that the test is still being used to date in research settings [106]. However, it is not feasibly to process large numbers of samples or (routinely) incorporate this technique in a diagnostic pathway. Similarly, western blot is being applied in research settings to investigate the presence of proteoforms in clinical samples as well as in recombinant study samples (de la Morena‐Barrio et al. 2022a). Similar to immunoelectrophoresis it is a labor‐intensive technique but does provide insight on the number of proteoforms present in a sample, provided that they can be separated on gel (which may be troublesome for point mutations leading to very small mass differences). Western blot does enable information on different proteoforms due to altered mass or glycosylation, although it does not reveal which exact proteoform is present. For instance, western blot may identify variant proteoforms with an altered isoelectric point, in the laten conformation or showing altered glycosylation. The latter is based on mass shifts corresponding to the mass of a glycan or by applying de‐glycosylating enzymes such as PNGase F as a control (de la Morena‐Barrio et al. 2016) which indicates altered overall glycosylation but not the exact sites at which glycans may be absent. However, studies investigating glycoproteoforms did enable valuable insight into the clinical relevance of AT glycosylation, albeit translation of this information into the diagnostic and clinical pathway has remained troublesome (de la Morena‐Barrio et al. 2016, 2022a). Lastly, ELISA may also be applied to investigate the concentration of AT in samples, although this is the least informative research technique providing merely a concentration without information on the proteoforms. Furthermore, this technique is dependent on antibodies, which, combined with the indirect read‐out of ELISAs, may lead to errors (Matson 2023).

### 5.2 Progressive Antithrombin Activity Test

The standard AT activity test lacks sensitivity but has the benefit of being easily applied in large cohorts or for diagnostic purposes. Thus, in an attempt to increase its sensitivity and make the activity test more valuable, Kovács et al. established an alternative chromogenic activity test, termed the progressive AT test. The test makes use of AT's dependence on heparin and the existing infrastructure and protocols for the standard AT activity test. In short, polybrene, a heparin neutralizer, is added to the sample dilution buffer and the incubation time of the reaction mixture is extended, allowing examination of AT activity without heparin acting as a cofactor (Kovács et al. 2014). The ratio of the progressive test versus a heparin‐cofactor dependent test provides a ratio that discriminates between type I and type II ATD and could even distinguishing between homozygous and heterozygous p. Leu131Phe patients (a type II HBS deficiency). As the test requires a relatively simple adjustment of the standard activity test it may be executed in any laboratory that already executes AT activity tests to provide clinically relevant information in the absence of genetic screening (Van Cott et al. 2020; Cooper et al. 2011). However, the test does not provide detailed molecular information on the mutation underlying the ATD, nor the glycosylation status, and certain HBS mutations were found to still be missed with this test (Moore et al. 2015). Therefore, although currently a viable option to improve the ATD clinical care pathway while using the existing infrastructure, the progressive activity test is by no means a suitable candidate test to facilitate P5 healthcare.

### 5.3 Genetic Testing

Genetic testing is the technique most commonly mentioned when precision medicine is discussed, largely owing to the success of genomic profiling in precision oncology (Mateo et al. 2022). However, in the field of thrombosis and haemostasis, the introduction of genetic testing has been limited almost exclusively to research and has only been sparsely applied in clinical practice (Nagalla and Bray 2016). It has been suggested to selectively genetically screen patients suspected of ATD (Colucci and Tsakiris 2017; Van Cott et al. 2020), specifically for mutations that are known to present with normal activity and antigen values, such as transient hereditary AT deficiencies caused by AT Dublin or AT Cambridge II mutations (Bravo‐Pérez et al. 2022). Furthermore, genetic testing can determine homozygosity of a mutation. Zeng and coworkers even recommended the inclusion of genetic screening as a first‐line test upon suspicion of hereditary AT deficiency, due to the risk of missing diagnoses (Zeng et al. 2015). However, as the exact mutation often does not lead to a change in treatment strategy, genetic testing is only sparsely applied and not always available. More importantly, genetic information cannot predict the expression and secretion of AT proteoforms into the circulations, nor can it identify alterations in posttranslational modifications, such as the glycosylation of AT, even though the latter was found to be clinically relevant (de la Morena‐Barrio et al. 2016). Furthermore, structural variants commonly occurring in ATD may be difficult to identify and certain mutations could influence splicing, further complicating obscuring the effect a mutation may have on te amino acid sequence (de la Morena‐Barrio et al. 2022b; de la Morena‐Barrio et al. 2017). Thus, it appears that the hype surrounding genetic screening in oncology is not translated to the field of thrombosis and haemostasis and instead alternative molecular techniques should be evaluated.

### 5.4 Multiple‐Reaction‐Monitoring Mass Spectrometry

Precision medicine is based on the concept that clinical care should be tailored to the individual patient, thereby optimizing patient management. To accommodate a precision medicine approach, focusing merely on genetics does not suffice, as posttranslational modifications, physiology, and environmental influences introduce additional variation at the patient‐level (Duarte and Spencer 2016). Proteins, in contrast to genes, are modified by these additional factors and actively play a role in health and disease. Therefore, proteomics and specifically the measurements of proteoforms has been put forth as a more refined technique to understand human biology and improve disease diagnostics (Duarte and Spencer 2016; Melani et al. 2022). Currently, the measurement of proteins in clinical chemistry relies heavily on immunoassays, despite potential flaws such as discordance between tests, interference by auto‐antibodies, and hook effects (Hoofnagle and Wener 2009). Furthermore, immunoassays offer limited insight into proteins, as they often only allow quantitation but no molecular information. Instead, mass spectrometry (MS) is the most omnipotent technique as it enables quantitation, identifies mutations, and monitors posttranslational modifications. To this end, MS has been proposed as a potentially revolutionizing technique in the clinical laboratory and for the application of precision diagnostics (Hoofnagle and Wener 2009; Ruhaak et al. 2016). Specifically, multiple‐reaction‐monitoring (MRM‐)MS currently holds the greatest potential due to its high sensitivity and specificity, two critical qualities for the development and validation of clinical diagnostic tests (Kitteringham et al. 2009).

#### 5.4.1 The Principle of Multiple‐Reaction‐Monitoring

Quantitative protein mass spectrometry through MRM‐MS is based on the measurement of surrogate peptides of the protein of interest using so‐called “transitions,” which is the combination of precursor peptide *m/z* and the *m/z* of related fragment ions (Lange et al. 2008). In comparison to intact proteins, peptides are smaller and analytically less challenging analytes (Monaghan et al. 2013), which are formed by digestion of proteins by a sequence specific enzyme, most often trypsin. To quantify these peptides, triple‐quadrupole MS instruments are used, which filter for the precursor peptide mass‐to‐charge (*m/z*) value in the first quadrupole, fragment this peptide in the second quadrupole, and filter for the fragment ion *m/z* value in the third quadrupole. If carefully validated, this peptide/fragment pair, or transition, is essentially a fingerprint of the protein of interest and provides high selectivity, which may be further increased through quantitation of multiple (e.g. three) transitions per peptide of interest and combining MRM‐MS with liquid chromatography to monitor the transition of interest at a specific retention time (Aebersold and Mann 2016). The triple‐quadrupole instrument filters for the transitions of interest and only these are passed to the MS detector, thus limiting the chemical noise. Through this mechanism, high analytical sensitivity is achieved (Kitteringham et al. 2009). Mass spectrometry is not inherently a quantitative technique, as signal intensity of the instrument may fluctuate between measurements, but the addition of internal (peptide) standards, combined with the application of calibration curves allows for the correction of these fluctuations, thereby enabling accurate and robust quantitation (Strathmann and Hoofnagle 2011; LeBlanc et al. 2017). Beyond quantitation, transitions may be developed that target mutations instead of wildtype peptides or include posttranslational modifications such as glycosylation (Kitteringham et al. 2009; Hong et al. 2013; Lin et al. 2023; Kreft et al. 2024). This entails that MS‐based tests can generate both quantitative and qualitative information, which is ideal to accommodate precision diagnostics.

#### 5.4.2 Applying MRM for the Advancement of Clinical Proteomics

Thus far, monitoring of mutations or posttranslational modifications by MS has mostly been applied in discovery proteomics and only sparsely in clinical proteomics. Even the translation of protein biomarkers to quantitative clinical chemistry proteomics faces many challenges, such as analytical performance and test robustness, clinical performance and clinical effectiveness (Lehmann et al. 2012). Therefore, extensive validation is required, ideally according to clinical chemistry guidelines. Even for the singleplex quantitation of an individual protein concentration by MS, this process and the associated quality control may already be challenging (CLSI 2021; Vogeser and Seger 2016). Ideally, a new test should be developed according to the test evaluation framework proposed by the EFLM to ensure that the test provides added benefits to the patient, either in the form of improved health outcomes or other aspects such as reduced time to diagnosis or lowered costs (Horvath et al. 2014). By adhering to this framework, it has been shown that when a test has been thoughtfully developed, validated according to predefined analytical performance specifications and the performance is continuously monitored, long‐term robustness of quantitative MS‐based tests is possible (Smit et al. 2021). For proteins existing in diverse proteoforms with clinical relevance, this strategy could be expanded to target mutated and modified peptides, thereby pioneerig into the new clinical chemistry field of quantitatively and qualitatively measuring proteoforms to enable precision diagnostics. Antithrombin is a prime example of a protein existing in clinically relevant proteoforms, for which the current clinical tests are underperforming, and to which end an MS‐based diagnostic proteoform test may be the key to unlocking a precision medicine approach.

#### 5.4.3 The LC‐MRM‐MS Test for Antithrombin

**Figure 5:**
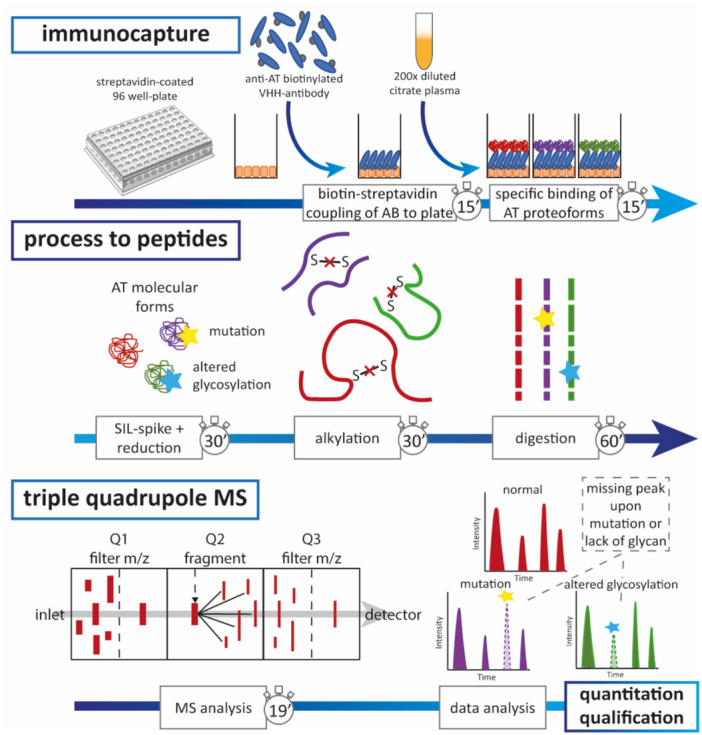
Overview of the steps of the LC‐MRM‐MS test for analyzing AT proteoforms. Stopwatches indicate time required per step for a single batch (of 96 samples), except for MS analysis, which takes 19 min per sample. [Color figure can be viewed at wileyonlinelibrary.com]

To address the current unmet clinical needs of AT diagnostics, an MS‐based test measuring AT proteoforms was developed within our clinical chemistry laboratory. An overview of this LC‐MRM‐MS test for AT is provided in Figure 5. An initial immunocapture step concentrates AT proteoforms from 200 times diluted patient plasma. It must be noted that the immunocapture step could introduce a bias towards specific proteoforms, although this has not been observed thus far careful evaluation of results is required (Kruijt et al. 2023). The low sample volume required (10 µL of citrate plasma), combined with high stability of proteoforms in LC‐MRM‐MS analysis, facilitates both prospective studies with minimal blood draw requirements as well as retrospective studies in cohorts with small volume bio‐banked samples. After immunocapture, stable isotope labelled (SIL‐) peptides are added as internal standard and the isolated AT proteoforms are denatured. The cysteine residues are reduced to break intra‐protein disulfide bonds, which are then irreversibly obstructed by alkylation. Lastly, AT proteoforms are digested with trypsin for 1 h, which is relatively fast owing to the immunocapture step that isolates AT from other proteins interfering with digestion. An optimized liquid chromatography gradient separates the tryptic peptides before entering the MS to enhance analytical sensitivity. Three precursor/fragment combinations are monitored per peptide in MRM‐MS for a total of twenty‐three wildtype AT peptides. Of note, all sample processing steps have been semi‐automated on a liquid handling platform.

The concentrations of twenty‐three wildtype AT peptides by themselves do not provide an easily interpretable diagnosis and therefore additional data handling is required. First, three quantifying peptides are used to confirm the overall AT proteoform concentration, which is reported by the concentration of a single representative quantifier peptide. The remaining twenty‐two peptides are then divided by this overall AT concentration, resulting in a ratio approximating 1 if only wildtype proteoforms and therefor wildtype peptides are present (with fluctuations from 1 occurring due to analytical variation). However, if a variant proteoform is present harboring a mutation or hypoglycosylation, the corresponding wildtype peptide will have a lower (or in specific cases higher) ratio than expected or would even be 0 in the case of a homozygous mutation. By comparing the ratio to defined limits, a stretch of protein harboring a mutation can be identified. Additional MS analysis with variant peptide specific transitions enables identification of the variant peptide by targeting the possible aberrations of that protein stretch. As such, mutations can be identified down to the amino acid change. Finally, a bi‐level diagnosis may be reported, informing on the overall AT concentration, allowing identification of quantitative ATD, and the presence of variant proteoforms, allowing identification of qualitative ATD down to the mutation (Kruijt et al. 2024). The data analysis is executed by a tailor‐made R script to enable an efficient data processing workflow. Thus, although the MS technique and subsequent data analysis are fairly complicated, the automation of both the sample processing as well as data handling facilitates application of the test in a clinical laboratory.

The application of the MS‐based test has already led to new insights into ATD, which could not have been generated using traditional or other molecular based techniques. An investigation by Kruijt et al. into the proteoforms composition of ATD patients using the MS‐test showed the large molecular heterogeneity of proteoforms composition within type II ATD patients (Kruijt et al. 2024). For example, the levels of AT proteoforms in type II ATD patients were found to vary between 16% and 58%. Furthermore, plasma samples of certain type I ATD patients, traditionally assumed to only have lower concentrations of AT, were found to also harbor variant proteoforms. Although further investigation into the effect of variant AT proteoforms on disease is required, the current classification of ATD into a purely quantitative or qualitative deficiency may no longer be suitable.

#### 5.4.4 Alternative Applications and the Future of the LC‐MRM‐MS Test for Antithrombin

Beyond the use of the MS‐based test to diagnose ATD in patients suspected thereof, the detailed investigation of antithrombin could also be applied in other clinical settings. This is owed to the unambiguous and direct monitoring of the protein. For instance, it was found that patients with sepsis have higher levels of alternative AT proteoforms (Mushunje et al. 2004). Another possible application could be the monitoring of AT levels and proteoforms in patients under anticoagulant treatment, to assess risk of recurrence. Of note, fitusiran, an siRNA designed to lower AT levels, is currently in the final phase of investigation for use as a treatment for haemophilia A (Srivastava et al. 2023). Monitoring of the AT levels by MS as a companion diagnostic in patients receiving fitusiran could be useful for both assessing the concentration of AT and monitoring the effect of fitusiran on the AT proteoform composition. Lastly, it could be imagined that other disorders affecting the composition of AT proteoforms or leading to modifications of the protein could be monitored using this test. As the MS‐based platform may easily be adjusted, one could include transitions to monitor other additional modifications beyond the currently included glycosylation if new modifications are deemed or anticipated to be clinically relevant.

This MS‐based molecular test is currently the most technologically advanced test of the repertoire, and monitors all molecular aspects anticipated to be of clinical relevance. Furthermore, the test has been analytically validated enabling application of the test in both a research setting for investigating large cohorts, as well as in a diagnostic setting for routine patient diagnostics as well as other clinical applications concerning antithrombin. However, MS is currently not a widespread technique within clinical chemistry laboratories, and for robust performance qualified personnel and specialized instrumentation is required, only available in the more specialized clinical chemistry laboratories. However, similar to the rise of genetic screening, the increasing application of MS is anticipated to result in more widespread expertise, ready to use applications and lowered costs of instrumentation, In the current situation, the establishment of centralized expertise centers is likely already sufficient to provide broad access to the next generation AT diagnostics MS test. As thrombophilia workup is only sparsely requested, a centralized laboratory would also ensure sufficient sample numbers to execute the test on a frequent and cost‐effective basis. Consequently, the LC‐MRM‐MS test offers the largest potential of all available alternative testing options to improve the current clinical pathway and enable a P5 healthcare approach for ATD.

## 6 Bringing Antithrombin Diagnostics Into the 21st Century

### 6.1 The Unmet Need in Antithrombin Testing

The current testing strategy for ATD relies heavily on AT activity tests, although these tests are imperfect in correctly diagnosing all ATD patients, and not seldomly leave clinicians questioning an ATD diagnosis. The introduction of the first AT activity test in the 1970s opened the valuable option to routinely screen suspected patients for (hereditary) ATD. Since then our knowledge of AT has grown to differentiate subtypes of ATD and observe that, even within these subtypes, diversity between ATD patients exists. AT activity tests have not developed along to reflect this. Consequently, the tests cannot differentiate specific ATD subtypes, not seldomly provide incorrect results for ATD caused by type II HBS variants, and do not allow stratification of individual patients for preventive therapy. Consequently, there is an inadequately performing component in the clinical pathway for ATD and therefore an unmet need for alternative (molecular) tests for diagnosing ATD, as already proposed by other groups (Kristensen et al. 2007; Roberts et al. 2022; Colucci and Tsakiris 2020; Marco‐Rico and Marco‐Vera 2023; Corral and Vicente 2015).

To effectively develop novel laboratory tests that increase medical value and resolve truly unmet clinical needs, it is important to first evaluate how the test should be applied to improve the testing strategy (Horvath et al. 2014). Therefore, the test purpose and test role should be defined. To improve the diagnostic efficiency of the ATD clinical pathway and move from a reactive approach to a preventive approach, a diagnostic test is required that investigates all clinically relevant aspects of AT. Based on current evidence, as presented in this review, this entails that it is valuable to know the exact molecular makeup of the AT protein, which includes possible mutations and posttranslational modifications. The sole execution of an activity test giving an indication on the functionality of the protein does not suffice; instead, we should resort to molecular techniques. Gene sequencing is currently the technique of choice for molecular diagnostics (Pei et al. 2023). The simple interpretation, low costs and fairly good availability has led to suggestions of applying gene sequencing for certain ATD patients or even for screening (Van Cott et al. 2020; Zeng et al. 2015). However, gene sequencing does not ascertain the expression of specific genetic variants resulting in their presence in the circulation, nor does it monitor the presence of posttranslational modifications. Consequently, focus is shifting from genetic to proteomic analysis to unravel the etiology underlying diseases (Smith et al. 2021b). As AT genetic variants may or may not be excreted in the circulation, and posttranslational modifications are important for AT functionality, proteomic analysis is the most appropriate technology to fully explore the molecular characteristics of the protein (Kruijt et al. 2023).

Molecular diagnostics of ATD at the proteomic level should ideally first provide an unambiguous diagnosis of ATD for all patients. Such diagnosis will allow every patient with ATD to receive treatment and empower patients in having an explanation for otherwise unexplained clinical symptoms such as VTE and recurrent pregnancy loss. However, beyond an accurate ATD diagnosis, a protein based molecular ATD diagnosis could enable risk stratification of specific thrombotic complications at an individual patients' level. While the low incidence of specific individual AT genetic variants thus far hampered the identification of variant specific (thrombotic) symptoms, evidence for variant specific stratification has been reported. For instance, the p. Pro73Leu mutation and the p. Leu131Phe mutation have been connected to increased incidence of arterial thrombosis as well as obstetric complications (Puurunen et al. 2013; Gindele et al. 2016). It is anticipated that a molecular AT test will enable further studies towards variant specific symptoms, and their risk profile, likely also providing evidence for variant specific optimal therapeutic strategies, required for P5 medicine (Anaya et al. 2016).

While ideally a test provides 100% clinical accuracy, the presence of analytical‐ and consequently clinical inaccuracy has to be accepted. Guidelines for acceptable measurement error have been developed by the European Federation of Clinical Chemistry and Laboratory Medicine (EFLM) and culminated in the Milan consensus on the deduction of analytical performance specifications (APS) based on three‐levels (Sandberg et al. 2015). Most ideal is the deduction of APS based on clinical outcomes (Horvath et al. 2015; Smith et al. 2019; Horvath et al. 2024). However, empirical data from randomized controlled trials that directly address the impact of variable analytical performance on clinical outcomes is not available and simulation models to address the impact of analytical precision on patient classification have not yet been developed for AT. The second order of data for the deduction of APS is by biological variation. As mentioned previously biological variation in AT may be caused by gender and age as well as hormone status. Based on activity tests, a within subject CV (CVi) of 3.4% and a between subject CV (CVg) of 7.8% were reported. Through application of the concept of Total allowable error (TEa) (Fraser 2001), a desirable imprecision and bias of 1.7% and 2.1%, respectively can be deduced, culminating in a TEa of 4.9%. Larger error allowances have been reported for AT concentration: CVi of 7.2% and CVg of 5.0% result in desirable imprecision of 3.6%, bias of 2.2% and TEa of 8.1% (Tables 1 and 2).

### 6.2 Next‐Generation Antithrombin Diagnostics by Mass Spectrometry

We developed a novel mass spectrometry‐based AT diagnostic lab‐developed‐test that fulfills the current requirements for in‐house IVDs according to the EU IVDR 2017/746 and ISO15189 (Kruijt et al. 2023). Through quantitation of 23 characteristic peptides originating from in‐vitro proteolytic digestion of AT, an individual's molecular composition of AT proteoforms can be deduced from three layers of information (Kruijt et al. 2023). First, the total AT protein concentration can be derived; second, variation in the amino acid backbone of AT, caused by genetic variants is evaluated and third the AT glycosylation status is assessed. The test generates a relatively large amount of complex data that is not immediately interpretable for laboratory specialists and clinicians. Therefore, a data analysis strategy was developed and implemented through a semi‐automated R script that provides an easily interpretable bilevel result comprising the AT concentration (in µmol/L) relative to its reference intervals as well as the absence or presence of molecular variants (Kruijt et al. 2024).

The test was analytically validated showing an average imprecision of 5.9% over a linear measuring range of 0.08 to 2.58 μmol/L (Kruijt et al. 2023). The test was not prone to interferences and in a method comparison to the STA®‐Stachrom® AT III Test activity test, a Pearsons R of 0.88 was obtained. Therefore, it was concluded that the test fulfilled the predefined analytical performance specifications. Subsequently, scientific validity was investigated in a cohort of 91 patients with ATD caused by various *SERPINC1* mutations. The test showed improved diagnostic performance compared to activity tests and equal performance compared to genetic sequencing (Kruijt et al. 2024), thus providing first evidence into the scientific validity of next‐generation AT diagnostics by MS to fulfill the clinical performance required to address the current unmet clinical need in AT testing. Successful application of the test in a diagnostic setting was also described in a case report of a woman with recurrent pregnancy loss and borderline low AT activity values.

As outlined in the EFLM framework test evaluation, a test has to be evaluated for analytical performance, clinical performance, clinical effectiveness, cost effectiveness and broader impact (Horvath et al. 2014). While the first has been accomplished, and the second has been initiated through the scientific validity study (Kruijt et al. 2024), the other aspects are yet to be investigated. It has to be noted that the incidence of ATD is relatively low (Tait et al. 1994; Wells et al. 1994), with specific genetic variants fulfilling the definition of a rare disease (diseases that affects no more than 1 in 2000 persons (Richter et al. 2015)). Consequently, typical study designs developed to address clinical performance and clinical effectiveness will likely have to be modified to accommodate the low incidence numbers, as has also been done in RCTs towards therapies for rare diseases (Pizzamiglio et al. 2022). As the analysis of proteoforms by LC‐MRM‐MS is currently the most omnipotent technique to investigate ATD, further investigations into the clinical performance and effectiveness of the test are warranted to ascertain its value for improving the clinical pathway for ATD.

## 7 Considerations for Implementation of Next‐Generation Antithrombin Diagnostics

### 7.1 Metrological Traceability of Antithrombin Tests and Reporting Units

As the world has progressed to a global economy with mobile societies uniform clinical decision limits in clinical care guidelines are key to enable generalization of the outcomes of clinical trials (Plebani and Lippi 2023). Ideally medical test results should be traceable to highest order internal standard within allowable measurement uncertainty and expressed in SI units as outlined in ISO guideline 17511 (ISO 2020). While a WHO reference standard (NIBSC code 08/258) for AT is available, its properties have not been evaluated according to the latest metrological insights and its value has been assigned in IU (Hogwood et al. 2010). Mass spectrometry is a technology that measures molar concentrations of analytes relative to a molar calibrator. Expression of protein concentrations generated by MS using percentage units is inherently metrologically wrong; therefore, it was decided to value assign the calibrators of the MS‐based test in µmol/L by converting the concentration in mg/mL of a Hyphen BioMED standard that is traceable to the WHO standard using the molecular weight of antithrombin (57855 kD, as assessed by intact mass spectrometry). It has to be noted that the mass spectrometry technology is suited to establish reference measurement procedures with unequivocally defined molecular measurands (Hoofnagle et al. 2021; Ruhaak et al. 2023). Provided molar value assigned reference materials become available it may be anticipated that the MS‐based AT test may play a role in standardization of commercial AT test results.

### 7.2 Clinical Care Guidelines

Beyond the availability of diagnostic tests, recommendations regarding thrombophilia testing in general are restricted to certain patient groups (Middeldorp et al. 2023; Arachchillage et al. 2022). The most recent guidelines from the American Society for Hematology (ASH) published in 2023 have omitted thrombophilia testing in patient groups such as patients with unprovoked VTE or surgery provoked VTE (Middeldorp et al. 2023). The ASH guidelines were established based on limited historical data on thrombophilia prevalence and relative risks for VTE recurrence in thrombophilia positive versus thrombophilia negative patients. Based on various factors such as the observed identification rate of thrombophilia's, possible treatment strategies and costs of performing a thrombophilia panel, the added benefit of knowing which thrombophilia was underlying a VTE was often negligible. Thus, for many scenarios it was recommended that it was better to provide a generalized treatment based on the clinical scenario than first perform a thrombophilia screening. However, as also noted by Cohen et al (Cohen et al. 2024)., the complex roles between thrombophilia testing and VTE management are overlooked when applying such generalized models.

For ATD specifically, a major flaw of these generalized approaches is that they are based on data from studies which identify patients as ATD using an in vitro activity test. This could lead to an underestimation of the AT prevalence and underrepresentation of specific subtypes in these studies (Orlando et al. 2015; Kovács et al. 2013). Importantly, subtypes that are currently underdiagnosed by activity tests do lead to increased VTE risks (Orlando et al. 2015), and their underrepresentation is therefore a critical flaw in our attempts to understand ATD diversity and provide optimal patient care. Even if these studies did include all individuals with ATD, a generalized treatment strategy based on the general prevalence and VTE risk of ATD is an extremely blunt and insensitive approach given the high diversity between patients, potentially leading to overtreatment for ATD with a mild clinical phenotype which brings along bleeding risk. Given the difference in functionality between AT proteoforms (Gindele et al. 2021), subtype or proteoform specific treatment strategies or even personalized medication are more fitting approaches compared to prescribing a general anticoagulant.

Lastly, and perhaps most importantly, the current clinical care pathway for ATD as outlined in the clinical care guidelines is based on a reactive disease approach, as highlighted by the ASH guidelines which assume that patients first present with a VTE before any form of screening or treatment is applied. With our focus on hereditary ATD, it is a fact that each ATD patient has the potential to be discovered at any point in life, also well before any clinical event has occurred. Such a strategy would open the possibility to provide prophylactic therapy even before the occurrence of a first thrombotic event.

### 7.3 Anticipated Test Role

**Figure 6:**
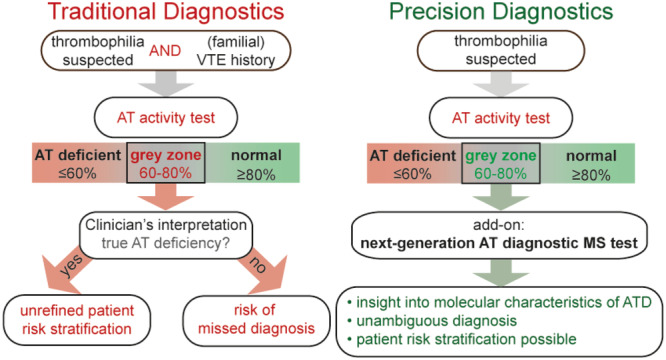
Clinical care pathways for ATD based on traditional (left) and Precision Diagnostics (right). The current clinical pathway is built upon imperfect AT activity tests, that carry the risk of missed diagnoses and do not allow patient risk stratification. Consequently, the target population is limited to individuals that are at high risk of ATD. The inclusion of a next generation AT diagnostic test by MS provides unambiguous diagnosis of ATD and consequently allows broadening of the target test population, eventually resulting in more patients being diagnosed and treated more accurately. Adapted from Kruijt et al. (2022). [Color figure can be viewed at wileyonlinelibrary.com]

The next‐generation AT diagnostics MS test is expected to be used as an add‐on test in conjunction with the AT activity tests for reducing the diagnostic uncertainty of the latter (Figure 6). Mass spectrometry technology is relatively complex and has only recently been introduced in the clinical chemistry setting (Annesley et al. 2016). Moreover, running costs of MS‐based tests are still high, requiring large upfront investment and qualified personnel, and throughput of MS‐based tests employing LC separation is relatively lower than that of automated coagulation instruments. The throughput of the next‐generation AT diagnostics test is ~150 samples per 24 h. As a result, mass spectrometry technology is currently only available in the diagnostic setting in specialized (mostly academic) clinical chemistry laboratories. While the increasing number of applications being described and the outlook on automated MS analyzers becoming available for easy integration in a diagnostic laboratory, provide the promise of reduced costs, easier applications and higher throughput (Jannetto and Fitzgerald 2016), it seems currently not realistic nor cost‐effective to replace activity tests with next‐generation AT diagnostics by MS. Rather, we envision the application of our MS based test to be complementary as an add‐on test to follow‐up individuals with low or borderline activity test results to ascertain the diagnosis, provide better patient stratification and generate more evidence into the molecular characteristics of ATD.

### 7.4 Future Clinical Pathway

**Figure 7:**
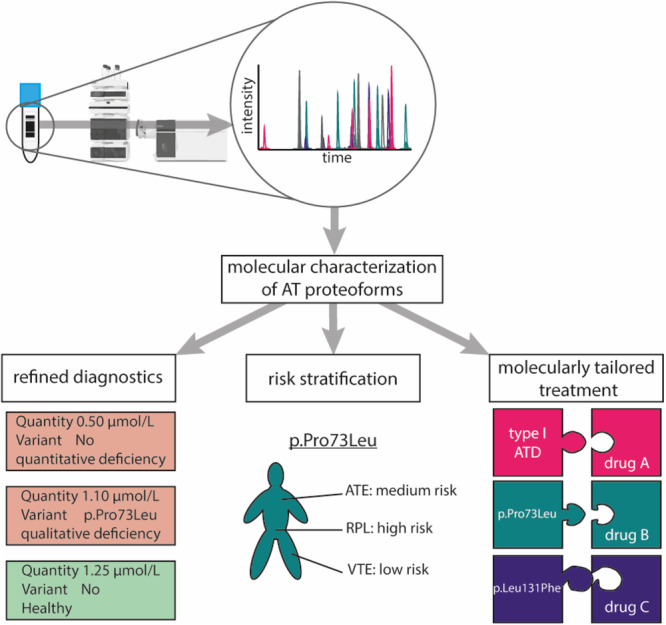
Overview of the capabilities of the LC‐MRM‐MS test for AT in a future clinical pathway. The test, performed on a triple quadrupole mass spectrometer using multiple reaction monitoring, allows for refined diagnoses at the molecular ATD level. It is anticipated that this will enable risk stratification based on the molecular diagnosis, as well as tailored (preventive) therapy. [Color figure can be viewed at wileyonlinelibrary.com]

The current clinical pathway has two main flaws, that are exemplary for our reactive healthcare system. First, AT diagnoses are missed and secondly, ATD patients are not well stratified according to clinical risks. Consequently, generic treatment is given only once a thrombotic event has occurred. The developed next‐generation AT diagnostics test is expected to improve the clinical pathway by both increased diagnostic accuracy and molecular diagnoses that are suitable for clinical risk assessment and prophylactic therapy (Figure 7). Currently, AT testing is ordered upon a thrombotic event (e.g. VTE) under specific provoking conditions, in asymptomatic individuals with a family history of VTE and known thrombophilia status or in asymptomatic individual with first degree relatives without a history of VTE that have ATD (Middeldorp et al. 2023). This strategy is mostly based on the uncertainty of clinical relevance of borderline AT activity test results in the absence of a family history of VTE. However, implementation of the next‐generation AT diagnostics test with high diagnostic accuracy as an add‐on test minimizes the uncertainty; consequently, the target population is envisioned to be expanded to individuals suspected to have a thrombophilia (either through family history or clinical symptoms such as VTE or RPL). The relatively low prevalence of ATD, and low indication for screening allows application of the next‐generation AT diagnostics test in AT centers of expertise for follow‐up of low and borderline activity test results in this expanded population.

While the results of the activity tests used in the current clinical care pathway only provide a % activity which only allows for simplistic and not necessarily specific risk stratification and requires further testing to even differentiate between type I and type II ATD, the next‐generation AT diagnostics test directly provides a molecular ATD diagnosis (Kruijt et al. 2024). Evidence for risk stratification based on the presence of specific molecular AT proteoforms is currently scarce (Alhenc‐Gelas et al. 2017; Bravo‐Pérez et al. 2019), but it suggests that individuals with specific molecular forms are at higher or lower risk to develop thrombotic events, depending on their molecular AT variant. Consequently, application of the next‐generation AT diagnostics test likely enables risk stratification, which would allow therapeutic dosing strategies to be tailored towards an individuals' specific risk level. Current pharmaceutical interventions are generic, aimed to generally increase AT activity. However, specific molecular AT defects could potentially be better targeted using alternative strategies (e.g. treating a type II HBS ATD with heparin may be less effective). In this light, advancements in thrombophilia drug discovery and gene therapy could be highly interesting and should be followed with great care (Tang et al. 2022). Together with the expansion of the tested population to individuals that only have a family history of thrombophilia, but who have not yet suffered a first thrombotic event, risk and molecular defect specific therapy could reform ATD management and improve patient outcome by reducing diagnostic uncertainty.

## 8 General Conclusion

The diagnostic pathway of ATD, which is built on the level of knowledge that was available 50 years ago (Sas et al. 1975b), is based on the unrefined concept of an indirect measurement of the mixture of AT proteoforms, providing a single numerical value, the activity percentage. Multiple studies investigated the molecular basis of ATD and reported distinct molecular features in specific ATD types, thereby pleading for the introduction of molecular tests for ATD diagnostics and stratification. However, “*panta rhei,”* or the concept that everything is plastic and in constant movement, appears not to apply to the suboptimal and imprecise diagnostic AT activity tests; instead various academic groups have endeavored to improve the current clinical care pathway with new (molecular) diagnostic tests, such as the progressive activity test, application of genetic screening, and most recently an extensive mass spectrometry‐based test (Corral et al. 2018; Kovács et al. 2014; Kruijt et al. 2023). Mass spectrometry offers the unique ability to concurrently monitor AT at a quantitative, genetic, and posttranslational level. The insight generated by this test enables refined patient diagnostics, improved molecular understanding of ATD, and provides a basis for P5 medicine (Pravettoni and Triberti 2020; Anaya et al. 2016). The generated insight that molecular variants matter may even propel the improvement of IVD tests, for which studies are currently undertaken. Taken together, next‐generation precision diagnostics of AT by mass spectrometry offers valuable clinical insight into the diversity of AT proteoforms and is expected to play a large role in propelling ATD diagnostics and management into the 21st century.

## Author Contributions

**Mirjam Kruijt:** data curation, formal analysis, writing – original draft. **Christa M. Cobbaert:** conceptualization, writing – review and editing. **L. Renee Ruhaak:** conceptualization, funding acquisition, writing – original draft, writing – review and editing.

## Conflicts of Interest

The authors declare no conflicts of interest.
